# Non-redundant Functions of IL-6 Produced by Macrophages and Dendritic Cells in Allergic Airway Inflammation

**DOI:** 10.3389/fimmu.2018.02718

**Published:** 2018-11-26

**Authors:** Ekaterina O. Gubernatorova, Ekaterina A. Gorshkova, Olga A. Namakanova, Ruslan V. Zvartsev, Juan Hidalgo, Marina S. Drutskaya, Alexei V. Tumanov, Sergei A. Nedospasov

**Affiliations:** ^1^Engelhardt Institute of Molecular Biology, Russian Academy of Sciences, Moscow, Russia; ^2^Faculty of Biology, Lomonosov Moscow State University, Moscow, Russia; ^3^Department of Cellular Biology, Physiology, and Immunology, Autonomous University of Barcelona, Barcelona, Spain; ^4^Department of Microbiology, Immunology and Molecular Genetics, University of Texas Health Science Center at San Antonio, San Antonio, TX, United States

**Keywords:** HDM-induced asthma, eosinophils, neutrophils, mouse models, house dust mite (HDM)

## Abstract

Asthma is a common inflammatory disease of the airway caused by a combination of genetic and environmental factors and characterized by airflow obstruction, wheezing, eosinophilia, and neutrophilia of lungs and sputum. Similar to other proinflammatory cytokines, IL-6 is elevated in asthma and plays an active role in this disease. However, the exact molecular mechanism of IL-6 involvement in the pathogenesis of asthma remains largely unknown and the major cellular source of pathogenic IL-6 has not been defined. In the present study, we used conditional gene targeting to demonstrate that macrophages and dendritic cells are the critical sources of pathogenic IL-6 in acute HDM-induced asthma in mice. Complete genetic inactivation of IL-6 ameliorated the disease with significant decrease in eosinophilia in the lungs. Specific ablation of IL-6 in macrophages reduced key indicators of type 2 allergic inflammation, including eosinophil and Th2 cell accumulation in the lungs, production of IgE and expression of asthma-associated inflammatory mediators. In contrast, mice with deficiency of IL-6 in dendritic cells demonstrated attenuated neutrophilic, but regular eosinophilic response in HDM-induced asthma. Taken together, our results indicate that IL-6 plays a pathogenic role in the HDM-induced asthma model and that lung macrophages and dendritic cells are the predominant sources of pathogenic IL-6 but contribute differently to the disease.

## Introduction

Allergic asthma is a chronic inflammatory disease of the airways that occurs in response to inhaled allergens, such as pollen, house dust mites, and fungi. The incidence and the severity of chronic lung diseases, such as allergic asthma, are growing and affect between 200 and 300 million people worldwide. It is also associated with a significant mortality rate (1). Asthma is characterized by swelling and narrowing of the airways, infiltration of eosinophils to the lungs and activation of Th2 cells (2). Unfortunately, the initial cause that triggers most chronic and acute lung diseases remains unknown, and currently available therapies only ameliorate the symptoms, but do not cure the disease. Thus, there is a pressing need to identify new targets and develop novel therapies, especially, for those most severely affected.

IL-6 is an inflammatory cytokine with pleiotropic functions, ranging from hematopoietic regulation and tissue regeneration to the induction of chronic inflammation (3, 4), sustaining autoimmunity (5) and tumorigenesis (6). IL-6, like other inflammatory cytokines, is elevated in acute and chronic asthma, perhaps as a byproduct of the ongoing inflammation. However, recent studies (7–13) provide evidence that IL-6, rather than being critically involved in lung inflammation, is actually playing a key role in the pathogenesis of asthma. Therefore, IL-6 should be considered as a potential target for the treatment of this disease.

In recent years significant efforts were made to develop mouse models for allergic airway disease, since this would allow for the role of selected genes and gene products in asthma pathophysiology to be established (14). Although mouse models do not replicate human asthma exactly, the uncovered pathogenic mechanisms of allergic airway inflammation may be generally applicable to humans (15). The combination of various molecularly defined allergens found within the house dust mite (HDM) *Dermatophagoides pteronyssinus* is the most common trigger of allergic asthma worldwide (16). For example, HDM extract contains proteases, which cause local damage to the epithelium. Therefore, it directly activates the epithelium, and the resulting Th2 inflammatory cascade, characterized by the infiltration of Th2 lymphocytes, eosinophils, and mast cells, closely reflects the sequence of events observed in humans. Thus, HDM-induced asthma presents the most clinically relevant mouse model to date.

Despite the fact that a number of mouse and human studies implicated IL-6 in the pathogenesis of allergic asthma, the exact molecular mechanism allowing IL-6 to interfere with the lung functions, as well as, the major cellular sources of pathogenic IL-6 (17) remain largely unknown. In the present study, using clinically relevant low-dose (10 μg) acute HDM asthma mouse model (18, 19), we applied reverse genetics to document the active role of IL-6 in the pathogenesis of acute asthma and uncover non-redundant contributions from two important cellular sources of IL-6: macrophages and dendritic cells.

## Materials and methods

### Mice

IL-6 KO mice were generated by crossing IL-6 floxed mice (IL-6fl/fl) (20) with CMV-Cre mice (21). Mice with ablation of IL-6 in myeloid cells (Mlys-IL-6 KO) were generated by crossing IL-6fl/fl mice with Mlys-Cre knock-in mice (22). Generation of mice with IL-6 deficiency in CD11c^+^ dendritic cells (CD11c-IL-6 KO) has previously been described (23). Mice were genotyped by genomic PCR of tail DNA: primers for Mlys-Cre transgene Mlys1 5′-CTTGGGCTGCCAGAATTTCTC-3′, Cre8 5′-CCCAGAAATGCCAGATTACG-3′; primers for CD11c-Cre transgene CD11c-Cre F 5′-ACTTGGCAGCTGTCTCCAAG-3′, CD11c-Cre R 5′-GCGAACATCTTCAGGTTCTG-3′. Animals with age of 8–12 weeks were used for experiments. All manipulations with animals were carried out in accordance with recommendations in the Guide for the Care and use of Laboratory Animals (NRC 2011), the European Convention for the protection of vertebrate animals used for experimental and other scientific purposes, Council of Europe (ETS 123), and “The Guidelines for Manipulations with Experimental Animals” (the decree of the Presidium of the Russian Academy of Sciences of April 02, 1980, no. 12000-496). All animal procedures were approved by the Scientific Council of the Engelhardt Institute of Molecular Biology, Russian Academy of Sciences.

### Induction of asthma using HDM

Purified House dust mite (HDM) (*Dermatophagoides pteronyssinus*) extract (Greer Labs, US) was used in the experiments. For HDM-induced allergic inflammation, mice were anesthetized using mild anesthesia and sensitized intranasally (i.n.) using 1 μg HDM or with saline serving as a control. After 1 week mice were challenged daily for 7 consecutive days with 10 μg HDM or saline (i.n.). Forty-eight hours after the last HDM challenge mice were sacrificed for analysis.

### Isolation of tissue cells

The trachea was cannulated and bronchoalveolar lavage fluid (BALF) was obtained twice via lavage with 0.8 ml sterile PBS each time. Blood was drawn to screen for serum antibodies and cytokines.

Whole lungs were cleared of blood via ventricular perfusion of the heart with 0.9% NaCl. The lungs were excised and minced with scissors. Reproducible lung tissue dissociation in 5 ml/column of HEPES digestion cocktail (Collagenase D-100 mg/ml, DNAse I-20,000 U/ml, HEPES buffer-10 mM HEPES in PBS) was obtained by using the gentleMACS Octo Dissociator (Miltenyi Biotec, Germany) program “lung_01” (36 s, 165 rpr), and then incubated for 25 min at 37°C. After that samples were further dissociated with gentleMACS Octo Dissociator program “lung_02” (37 s, 2,079 rpr) and centrifuged at 300 g for 5 min at 4°C. The lung suspension and any remaining lung tissue chunks were pushed through a 70 μm filter and centrifuged at 300 g for 7 min at 4°C. Lungs were layered over Percoll (GE Healthcare, Sweden) (80/40%) gradient and centrifuged at 330 g for 25 min at 4°C without braking, and the cells contained in the interphase were recovered.

Lung (tracheobronchial) lymph nodes (lung LN), peripheral lymph nodes (per. LN) and spleen were dissected by passing cell suspension through a 100 μm filter (for lung LN) and a 70 μm filter (for per. LN and spleen). Samples were centrifuged at 300 g for 7 min at 4°C. Lymph node cells were resuspended in 0.25 ml 2% PBS/FBS. Splenocytes were resuspended in 1 ml of ACK lysis buffer, washed twice and resuspended in 1 ml PBS/FBS.

### RNA isolation and cDNA preparation

For RNA extraction lung samples were homogenized in TRK Lysis buffer [20 μL 2-mercaptoethanol per 1 mL GTC Lysis Buffer (OMEGA biotek, US)] using IKA T10 basic Ultra Turrax Homogenizer (Germany), then centrifuged at maximum speed for 5 min. RNA was extracted using the RNA isolation kit (E.Z.N.A.® Total RNA Kit I, USA). The concentration and purity of RNA was defined by absorbance measurements at 260 and 280 nm with a NanoDrop spectrophotometer (Thermo). RNA was reverse-transcribed into cDNA using M-MLV reverse transcriptase (200 U/μl).

### RT-PCR

cDNA was then used for real-time quantitative PCR using 7500 Real Time PCR System amplificator (Apllied Biosystems). A SYBR Green Master PCR mix was employed to amplify *Actb, Il17a, Gob5, Il4, Il5, Il10, Il10, Il33, Il13, Tgfb1, Tslp, Il6, Ifng, Muc5ac, Muc5b, Il1b* using gene-specific primers (Eurogene, primer sequences are shown in Table 1).

**Table 1:** Primers for qPCR analysis.

***Actb***	**F 5^′^-TAAAACGCAGCTCAGTAACAGTCC-3^′^**
	R 5′-CTCCTGAGCGCAAGTACTCTGTG-3′
*Il17a*	F 5′-GGACTCTCCACCGCAATGA-3′
	R 5′-GGACTCTCCACCGCAATGA-3′
*Gob5*	F 5′-ACTAAGGTGGCCTACCTCCAA-3′
	R 5′-GGAGGTGACAGTCAAGGTGAG-3′
*Il4*	F 5′-GGTCTCAACCCCCAGCTAGT-3′
	R 5′-GCCGATGATCTCTCTCAAGTGAT-3′
*Il5*	F 5′-AGCACAGTGGTGAAAGAGACCTT-3′
	R 5′-TCCAATGCATAGCTGGTGATTT-3′
*Il10*	F 5′-ATTTGAATTCCCTGGGTGAGAAG-3′
	R 5′-CACAGGGGAGAAATCGATGACA-3′
*Il33*	F 5′-TGCTCAATGTGTCAACAGACG-3′,
	R 5′-TCCTTGCTTGGCAGTATCCA-3′
*Il13*	F 5′-CCTGGCTCTTGCTTGCCTT-3′,
	R 5′-GGTCTTGTGTGATGTTGCTCA-3′
*Tgfb1*	F 5′-ACCATGCCAACTTCTGTCTG-3′,
	R 5′-CGGGTTGTGTTGGTTGTAGA-3′
*Tslp*	F 5′-TCGAGCAAATCGAGGACTGTG-3′
	R 5′-CAAATGTTTTGTCGGGGAGTGA-3′
*Il6*	F 5′-CTGATGCTGGTGACAACCAC-3′
	R 5′-GCCACTCCTTCTGTGACTCC-3′
*Ifng*	F 5′-TCAAGTGGCATAGATGTGGAAGAA-3′,
	R 5′-TGGCTCTGCAGGATTTTCATG-3′
*Muc5ac*	F 5′-AGAATATCTTTCAGGACCCCTGCT-3′,
	R 5′-ACACCAGTGCTGAGCATACTTT-3′
*Muc5b*	F 5′-TCCTGCTCTGGAATATCCAAG-3′
	R 5′-GCCTCGGGGAGCTTGCCTGCC-3′
*Il1b*	F 5′-CAACCAACAAGTGATATTCTCCATG-3′
	R 5′-GATCCACACTCTCCAGCTGCA-3′

Comparative CT method (2^−ΔΔ*Ct*^) was used. mRNA levels for the genes of interest relative to the expression of *actin beta* as housekeeping gene were obtained (ΔCt). ΔΔCt values were then obtained by subtracting the ΔCt value from a given reference sample as a calibrator to the rest of the samples. The mean of the ΔCT value within each group was used as a calibrator. The final relative expression data were obtained as 2^−ΔΔ*CT*^, defined as RQ value (relative quantitation).

### ELISA

The supernatants from BAL fluid and serum were collected for the measurement of cytokines and IgE using commercially available enzyme-linked immunosorbent assay (ELISA) kits (Invitrogen, Austria). IgE, IFNγ, IL-6, IL-13, and IL-1β levels in serially diluted serum and BALF samples were analyzed using HRP-conjugated antibodies specific for these cytokines. IgE levels in serially diluted serum and BALF samples (1:250 for serum and 1:20 for BALF) were determined with anti-mouse IgE as capture antibody (Invitrogen, Austria) and horseradish peroxidase (HRP)-conjugated anti-mouse IgE as detection antibody (Invitrogen, Austria), with mouse IgE as a standard (Invitrogen, Austria). Procedures were performed according to the manufacturer's instructions. Multiscan Go spectrophotometer (Thermo Scientific) was used to measure optical density at 450 nm, calculations of protein levels in serum and BALF were performed using SkanIt Software 4.0 (Thermo Scientific).

### Flow cytometry analysis

For flow cytometry, FcRs were blocked with Ab 2.4G2 (10 μg/ml), followed by staining with Abs against various surface markers. Myeloid cells were stained with Fixable Viability Dye-eFluor 780 (eBioscience), MHCII-PE (NIMR-4, eBioscience), CD11c-APC or CD11c-AmCyan (N418, BioLegend), CD11b-AmCyan or CD11b-PerCP-Cy5.5 (M1/70, eBioscience), Ly6G-FITC or Ly6G-Pacific Blue (RB6-8C5, eBioscience), Ly6C-PE-Cy7(HK1.4, eBioscience), CD45-PerCP-Cy5.5 or CD45-FITC (both 30-F11, eBioscience), F4/80-Pacific Blue or F4/80-PE-Cy7 (both BM8, eBioscience), SiglecF-PE (1RNM44N, eBioscience), CD103-APC (2E7, eBioscience). Lymphocytes were stained with Fixable Viability Dye-eFluor 780 (eBioscience), TCRβ-PE (H57-597, eBioscience), CD8-APC (53-6.7, BioLegend), CD4-PerCP-Cy5.5 (GK1.5, eBioscience), CD25-Pacific Blue (PC61.5, eBioscience), NK1.1-AmCyan (PK136, eBioscience), ST2-FITC (RMST2-2, eBioscience).

For intracellular staining of IL-6, cells were stimulated with 50 ng/mL phorbol myristate acetate (PMA) and 500 ng/mL ionomycin in RPMI 1640 medium supplemented with 10% fetal bovine serum (FBS) in a humidified atmosphere containing 5% CO_2_ at 37°C for 4 h. Brefeldin A was used to block protein transport to Golgi apparatus and accumulate proteins in the endoplasmic reticulum. Immediately after activation, cells were washed and stained for surface markers. Distinct populations of lymphocytes were distinguished with the following Ab panel: Fixable Viability Dye-eFluor 780, anti-CD45 (30-F11), anti-Ly6G (RB6-8C5), anti-CD103 (2E7), anti-CD11b (M1/70), anti-CD11c (N418), anti-SiglecF (1RNM44N), anti-CD4(GK1.5, eBioscience), anti-CD8(53-6.7, BioLegend), anti-ST2(RMST2-2, eBioscience), anti-CD19 (6D5, eBioscience) conjugated with FITC, PE, APC, PE-Cy7, PerCP-Cy5.5 and AmCyan. Cells were fixed in Permeabilization buffer and incubated at 4°C for 1 h. Myeloid cells and lymphocytes were washed, centrifuged at 400 g for 5 min and stained with anti-IL-6 PB-conjugated antibody (MP5-20F3, eBioscience) in 1X Permeabilization buffer at 4°C for 1 h. Mouse IgG1 Ab-PB (MOPC-21, eBioscience) was used as isotype control. Data were acquired using flow cytometer FACSCanto II (BD Biosciences). Data analysis was performed using FlowJo software (Tree Star).

### Histology

Lung tissue samples from each experimental group were fixed with 10% neutral buffered formalin solution and embedded in paraffin. After deparaffinization, sections of 4 μm-thickness were stained with periodic acid-Schiff (PAS) to identify degree of expression of mucosal glycoproteins. Slides were examined by light microscopy to evaluate the degree of airway inflammation.

### Statistical analysis

All experiments described were performed 2–4 times. Statistical analyses were performed using Prism 7 (GraphPad Software). Datasets were first tested for Gaussian distribution with the D'Agostino & Pearson omnibus normality test. Statistical significance was determined using Two-tailed, Mann-Whitney test or Two-way ANOVA, followed by Bonferroni post-test analysis for multiple comparisons. Results are expressed as mean ± SEM or ± Mean. *P* < 0.05 was considered statistically significant.

## Results

### IL-6 deficiency attenuates eosinophilic inflammatory response to *Dermatophagoides pteronyssinus* extract

Although IL-6 was implicated in the pathogenesis of allergic asthma both in human patients and in several mouse models of asthma (11, 24, 25), the contribution of this cytokine in the most clinically relevant mouse model—administration of HDM at low doses—has not been addressed.

**Figure 1:**
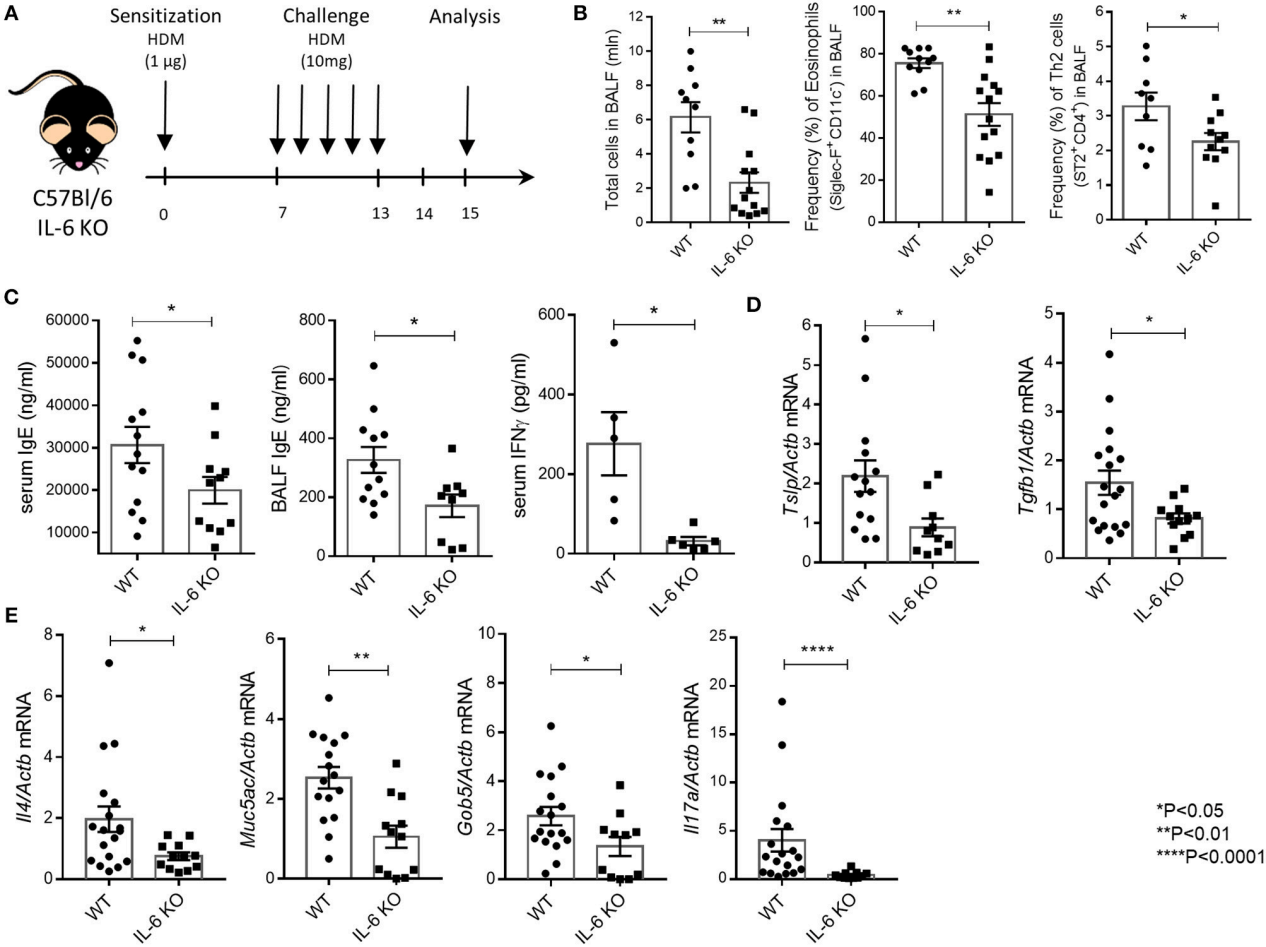
IL-6 deficient mice are resistant to HDM-induced asthma. **(A)** Scheme of the experiment. 8–12-weeks-old mice were sensitized intranasally with HDM extract (Greer Labs) on day 0 (1 μg of total protein in 10 μl). On days 7–13 mice were intranasally challenged with HDM extract (10 μg in 10 μl) and analyses were performed 48 h after the last challenge. All mice were euthanized by intraperitoneal injection of a lethal dose of the Zoletil/Rometar cocktail for anesthesia, and BAL fluid was collected. Lungs were then perfused by cardiac puncture using 10–15 ml DPBS, and lungs, spleens, and draining lymph nodes were harvested for RNA isolation and FACS analysis. **(B)** Total number of cells in BAL fluid and the frequency (%) of eosinophils (CD45^+^ SiglecF^+^ CD11c^−^) and Th2 cells (CD45^+^CD3^+^CD4^+^CD8^−^ST2^+^) in the BAL of WT and IL-6 KO mice assessed by flow cytometry. **(C)** Serum was collected 24 h after the last HDM challenge. The IgE levels in serum and BAL fluid and IFNγ levels in serum were determined by ELISA. **(D)** Quantitative RT-PCR analysis of *Tslp* and *Tgfb1* mRNAs in lungs 48 h after immunization. Expression of each gene was normalized to *Act*β. **(E)** Quantitative RT-PCR analysis of *Muc5ac, Gob5, Il4*, and *Il17a* mRNAs in lungs 48 h after last HDM challenge. Expression of each gene was normalized to *Actb*. Datasets were first tested for Gaussian distribution with the D'Agostino & Pearson omnibus normality test. Parametric or non-parametric comparison tests was applied where appropriated. Data represent means ± SEM, 5–17 mice in each group.

To investigate the role of IL-6 in allergic airway inflammation, acute asthma was induced in WT and IL-6 deficient mice by intranasally administering HDM extract−10 μg of protein for 7 days following sensitization with 1 μg of protein 1 week prior to the main course as shown on Figure 1A. Serum was collected 24 h after the last challenge, and 48 h after the last HDM administration mice were euthanized and BAL fluid, lungs, spleens, and draining lymph nodes were harvested for gene expression, cytokine production and FACS analysis (Figure 1 and Supplementary Figure 1).

Mice with genetic IL-6 deficiency mounted a significantly impaired airway inflammatory response as compared to WT mice. Flow cytometry of BAL cells revealed global decrease in inflammation, i.e., reduction in all cell types, in both the airway and lung tissue of IL-6 KO mice. Genetic ablation of IL-6 signaling was associated with abrogated infiltration of eosinophils and Th2 cells to the airways and the lung tissue (Figure 1B). This impaired inflammatory response was characterized by decreased numbers of lymphocytes in BAL fluid in IL-6 KO mice (Figure 1B). Furthermore, IL-6-deficient mice showed a marked decrease in IgE levels, a signature asthma antibody, in both BALF and serum as compared with their WT counterparts (Figure 1C). Interestingly, IFNγ level in serum was decreased in IL-6 KO mice as compared to WT mice (Figure 1C). Expression levels of TSLP, the master-regulator of airway remodeling during asthma, and of TGFβ1, which is involved in airway inflammation and hyper-responsiveness, were also decreased (Figure 1D) in IL-6 KO mice as compared to WT mice. Notably, inhibition of eosinophilic inflammation was associated with considerably lower expression levels of *Il4* (Figure 1E). Moreover, mucus production was reduced in mice with IL-6 deficiency, since the expression level of *Muc5ac*, the major airway mucin, and *Gob5*, a goblet cell marker, were significantly lower as compared to WT mice (Figure 1E). Finally, the expression level of *Il17a* was reduced in IL-6 KO mice compared to WT controls (Figure 1E). The inflammatory cells infiltrate around the bronchioles and vessels, as well as, the mucus layer (Supplementary Figure 3), were also reduced in IL-6 KO mice. These results are in accordance with earlier findings that IL-6 may enhance airway hyper responsiveness, allergic inflammation and development of airway remodeling in the high dose HDM-induced asthma model (24).

**Figure 2:**
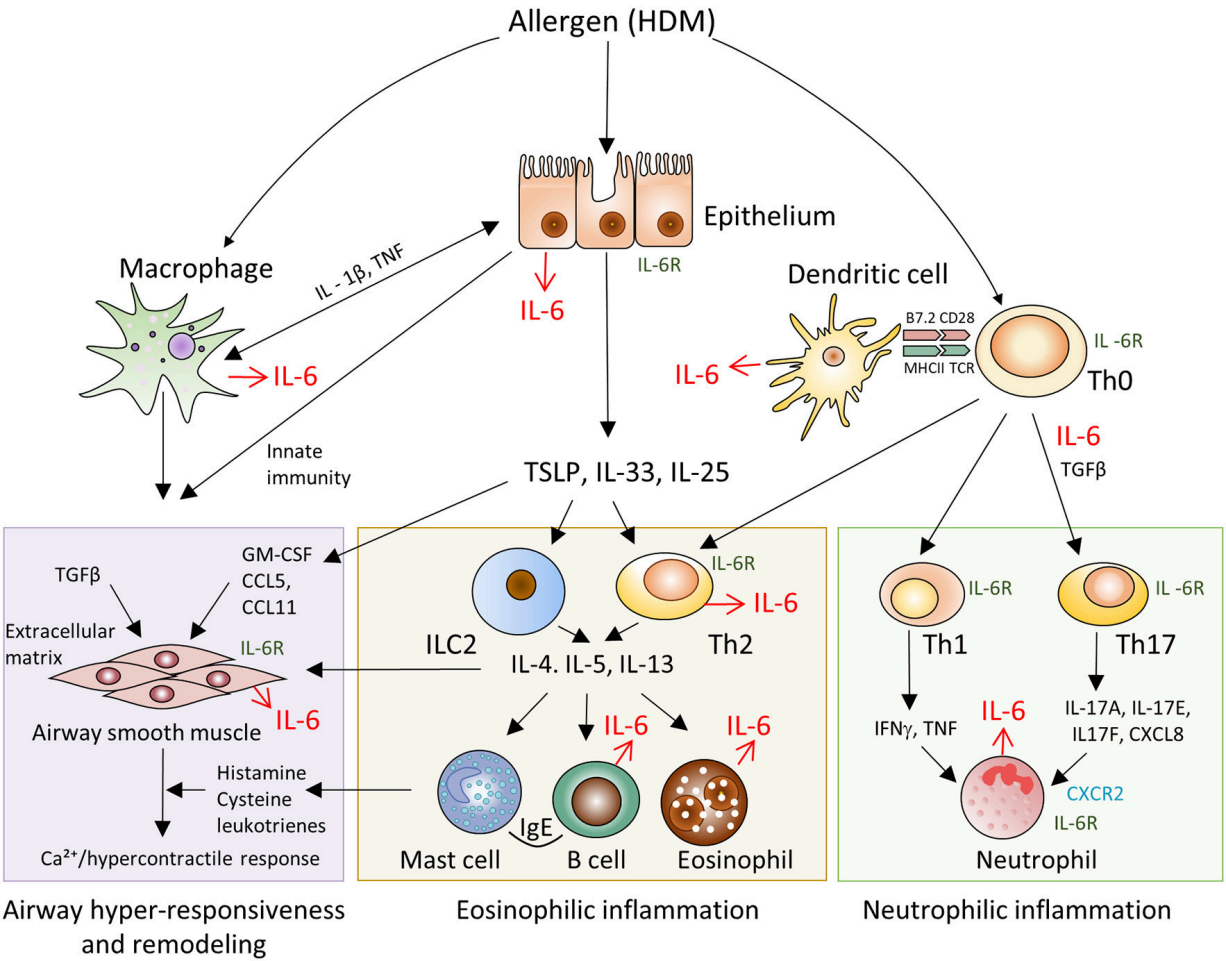
Schematic representation of IL-6 driven pathways and cell types involved in the pathogenesis of allergen-induced asthma. Pathogenesis of asthma is based on several interrelated processes and molecular cascades. Eosinophilic (Th2-high) airway inflammation (central panel) is present in more than 50% of adults with asthma and arises after sensitization to allergen. Eosinophilic asthma is characterized by Th2 cell activation, IL-4, IL-5, IL-13 production, high IgE levels and strong eosinophilia. Low-eosinophilic, neutrophil-predominant asthma (right panel) is less common, but often presents a more severe disease onset that does not respond to corticosteroid therapy. Airway hyper-responsiveness and remodeling (left panel) are the features present in all asthma subtypes.

Asthma is associated with a broad spectrum of clinical manifestations, ranging from mild to a severe disease onset, as well as, intractable disease. In general, pathogenesis of asthma is based on several interrelated processes and molecular cascades (Figure 2). Transcriptomic profiles of bronchoscopic samples led to identification of molecular phenotypes consistent with high type 2 immunity (26) and low type 2 immunity asthma (27). Eosinophilic (Th2-high) airway inflammation (Figure 2, central panel) is present in more than 50% of adults with asthma and arises after sensitization to allergen (28). Eosinophilic asthma is characterized by Th2 cell activation, IL-4, IL-5, and IL-13 production, high IgE levels and strong eosinophilia (29). Low-eosinophilic, neutrophil-predominant asthma (30) (Figure 2, right panel) is less common, but often presents a more severe disease that does not respond to corticosteroid therapy (31–34). Airway hyper-responsiveness and remodeling are the features present in all asthma subtypes (34).

IL-6 is known for its capability to promote differentiation of Th2 cells (35), to inhibit Th1 and Treg differentiation and expansion in response to allergen, to activate immunoglobulin class-switching in plasma cells and to enhance the differentiation of Th17 cells (36), which are engaged in amplification of severe asthma, and to control airway remodeling (37). Our observations in mice with systemic inactivation of IL-6 (Figure 1) provided a rational basis to address the impact of IL-6 from distinct cellular sources in clinically relevant low dose HDM-induced airway inflammation.

### Leukocytes are the major cellular source of IL-6 in the lungs at steady state and after HDM administration

**Figure 3:**
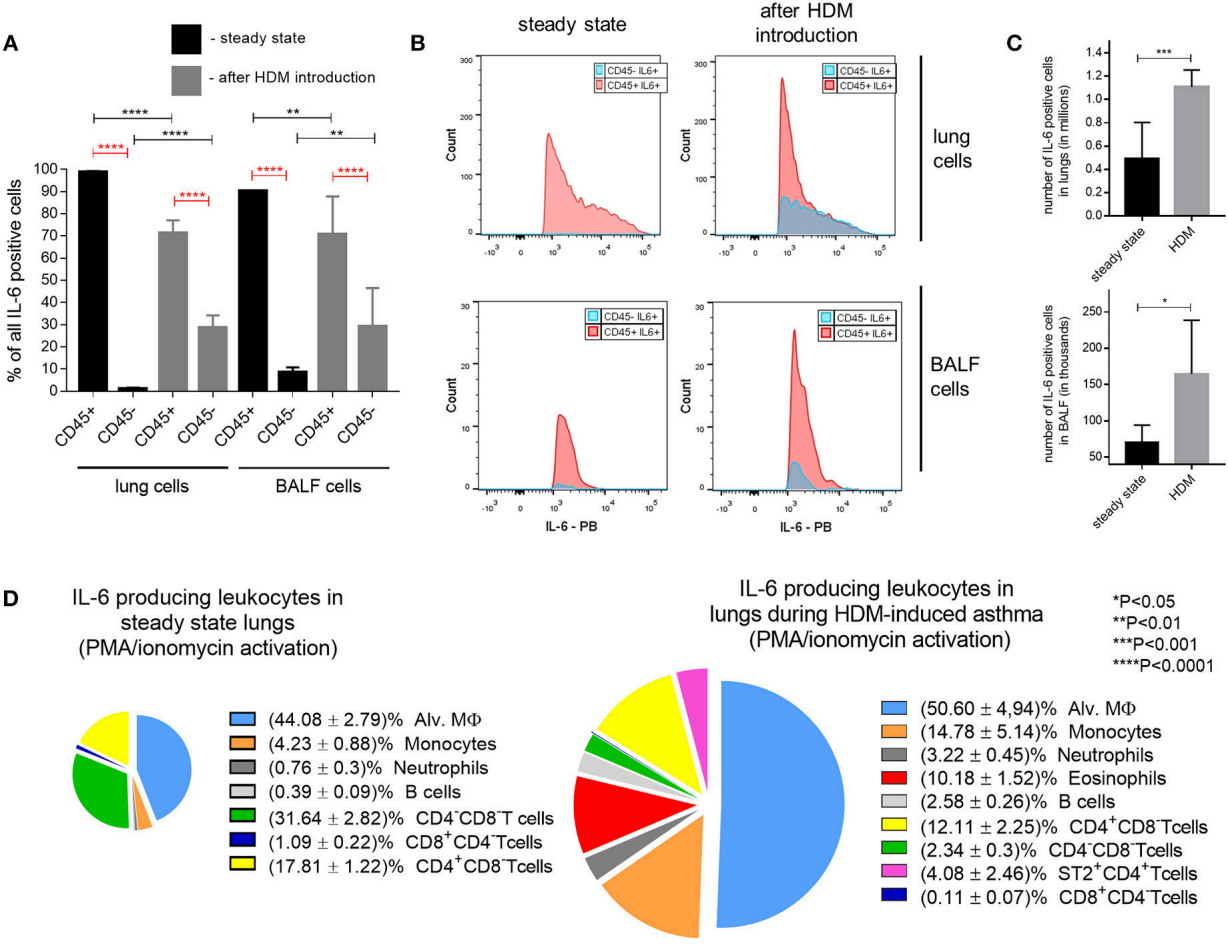
Cellular source of IL-6 in HDM-induced asthma. **(A)** Frequency (%) of lung and BAL fluid CD45^+^ (leukocytes) and CD45^−^ (non-leukocytes) IL-6 producing cells in steady state (black bars) and 48 h after last HDM introduction (gray bars) in WT mice assessed by flow cytometry. **(B)** Mean fluorescence intensity (MFI) of non-hematopoietic (CD45^−^, blue histograms) and hematopoietic (CD45^+^, red histograms) IL-6 expressing cells in lungs and BAL fluid in steady state and after HDM introduction. **(C)** Number of IL-6 producing cells in BALF and lungs in steady state (black bar) and 48 h after last HDM introduction (gray bar) in WT mice. **(D)** Representative percentage ratio of IL-6 producing cells of different lung leukocyte populations in steady state (left) and 48 h after last HDM introduction (right) in WT mice assessed by flow cytometry. Datasets were first tested for Gaussian distribution with the D'Agostino & Pearson omnibus normality test. Parametric or non-parametric One-way ANOVA with multiple comparisons tests was applied where appropriated. Data represent means ± SEM, 5–11mice in each group.

At steady state, IL-6 is produced by macrophages, dendritic cells, neutrophils, B cells and by some CD4^+^ T cells. In addition, IL-6 can also be secreted by endothelial cells, fibroblasts and epithelial cells (Figure 2). To determine the cell type that makes the most significant contribution to the production of IL-6 during asthma, we first examined whether the lymphoid or non-lymphoid cells produce higher amounts of IL-6 in steady state and in response to HDM. Allergic asthma was induced in wild-type mice in accordance with the protocol described above (Figure 1A) and mice receiving saline instead of HDM were used as a control group. Following 48 h after the last HDM injection, mice were euthanized, BAL and lung cells were isolated and analyzed for IL-6 by flow cytometry. Flow cytometry of BAL and isolated lung cells revealed that in steady state, as well as, in response to HDM, leukocytes (CD45^+^ cells) were the main source of IL-6 both in the BAL fluid and in the lungs (Figure 3A).

It should be noted that HDM administration resulted in the increase of a fraction of non-lymphoid IL-6-producing cells (Figure 3A). However, the percentage and fluorescence intensity of the IL-6-expressing lymphoid cell population remained significantly higher following asthma induction (Figure 3B). Immune cell counts as assessed by FACS analysis of IL-6 positive cell populations indicated that the absolute numbers of IL-6 positive cells sharply increased both in the airway and in the lung tissue of mice in response to HDM (Figure 3C), consistent with the notion that IL-6 is actively involved in the pathogenesis of allergic asthma.

To assess which leukocyte population is involved in IL-6 production in the low dose HDM asthma model, we induced asthma in wild-type mice, as shown on Figure 1A. Following 48 h after the last HDM injection, infiltrating lung lymphocytes were obtained and subjected to FACS analysis. The main sources of IL-6 in the lungs appeared to be monocytes, dendritic cells, macrophages, B cells, T cells and CD8^+^ T cells (Figure 3D; Supplementary Figure 2). Interestingly, at steady state conditions, most of the IL-6 producing cells were represented by macrophages and monocytes (i.e., myeloid cells), whereas after the induction of asthma, the contribution of the T cell fraction increased (Figure 3D). In summary, these results suggested that IL-6 from subtypes of myeloid cells, such as dendritic cells and macrophages, may play a pathogenic role in the development of allergic asthma.

### Mice with IL-6 deficiency in macrophages demonstrate attenuated Th2 response, eosinophilic inflammation and IgE production in HDM-induced asthma

To specifically address the role of IL-6 produced by macrophages in allergic airway inflammation, we generated mice with tissue-restricted inactivation of IL-6 in myeloid (Mlys-IL-6 KO) cells. It should be noted that, despite the fact that Mlys-Cre mediated deletion removes IL-6 not only in macrophages, but also in other myeloid cells (38), in our model neutrophils do not have any impact on IL-6 production either in steady state or after HDM is administered to wild-type mice, as shown in Figure 3D. Therefore, results obtained with Mlys-IL-6 KO mice, defined the contribution of IL-6 produced by macrophages in pathogenesis of acute HDM-induced asthma.

Mlys-IL-6 KO mice, as well as, IL-6 KO mice and littermate WT control mice, were subjected to intranasal administration of HDM extract for 7 days with additional sensitization treatment 1 week prior to the main course (Figure 1A). Serum was collected 24 h after the last HDM challenge, and the IgE in serum and in BAL fluid were determined by ELISA. Following 48 h after the last injection, mice were sacrificed and BAL fluid, lung infiltrating lymphocytes and splenocytes were collected for qPCR and FACS analysis.

**Figure 4:**
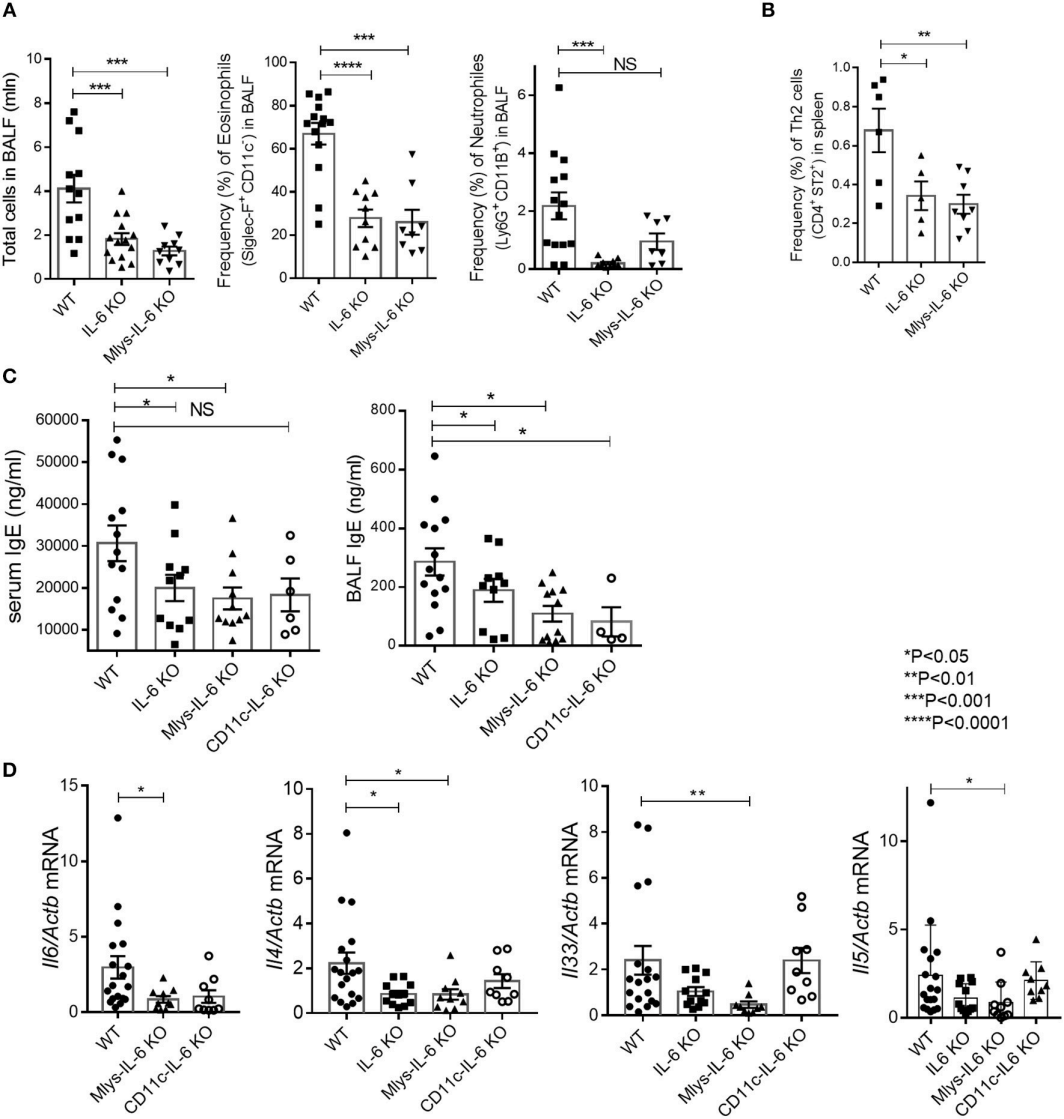
Mlys-IL-6 KO mice demonstrate attenuated type 2 response in HDM-induced asthma model. **(A)** Total number of cells in BAL fluid and the frequency (%) of eosinophils (CD45^+^ SiglecF^+^ CD11c^−^) and neutrophils (CD45^+^Ly6G^+^CD11b^+^) in the BAL of WT, IL-6 KO, and Mlys-IL-6 KO mice assessed by flow cytometry. **(B)** Frequency (%) of Th2 cells (CD45^+^CD3^+^CD4^+^CD8^−^ST2^+^) in the spleens of WT, IL-6 KO and Mlys-IL-6 KO mice assessed by flow cytometry. **(C)** Serum was collected 24 h after the last HDM challenge. The IgE levels in serum and BAL fluid were determined by ELISA. **(D)** Quantitative RT-PCR analysis of *Il6, Il4, Il33*, and *Il5* mRNAs in lungs 48 h after the last HDM challenge. Expression of each gene was normalized to *Actb*. Datasets were first tested for Gaussian distribution with the D'Agostino & Pearson omnibus normality test. Parametric or non-parametric One-way ANOVA with multiple comparisons tests was applied where appropriated. Data represent means ± SEM, 5–18 mice in each group. NS, not significant.

Unexpectedly, we found that genetic ablation of macrophage-derived IL-6 ameliorated the disease with significant decrease in the number of total BAL fluid lymphocytes and in the eosinophilia of the respiratory tract (Figure 4A). To establish whether the downshift in the local lung inflammation affected the systemic accumulation of Th2 cells, we determined the frequency of Th2 cells in the spleens of mice from different experimental groups. We found that the removal of IL-6 from macrophages not only reduced local inflammation in the airways, but also diminished the systemic type 2 response, previously characterized by the expansion of Th2 cells (Figure 4B). Moreover, Mlys-IL-6 KO mice displayed a significant reduction in IgE, a key indicator of atopy and of allergic inflammatory processes in the respiratory tract, in serum and in BAL fluid (Figure 4C). Quantitative RT-PCR analysis showed that the expression levels of *Il4*, a key mediator of Th2 response, and of the *Il33* gene, which is necessary for the development of allergic rhinitis, were reduced in the lungs of Mlys-IL-6 KO mice as compared to WT control mice (Figure 4D). Finally, we found that Th2, but not Th17, response was ablated in mice with IL-6 deficiency restricted to macrophages (Supplementary Figure 4). Taken together, these results suggested that macrophage-derived IL-6 plays a critical pathogenic role in the HDM-induced asthma model and that IL-6 from this cell type may contribute to the induction or amplification of Th2 inflammatory response during acute asthma.

### Mice with IL-6 deficiency in dendritic cells demonstrate attenuated neutrophilic, but regular eosinophilic response in HDM-induced asthma

**Figure 5:**
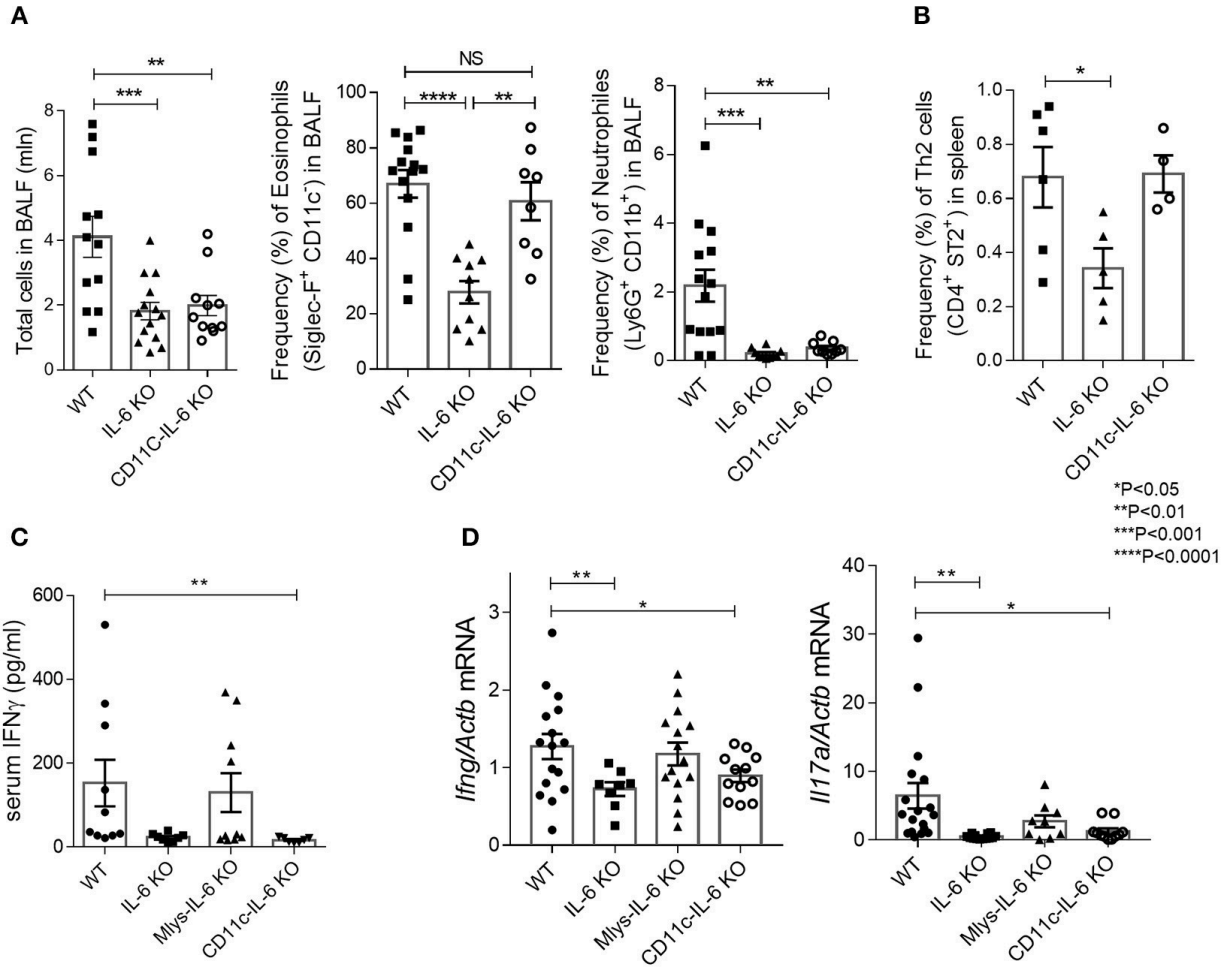
CD11C-IL-6 KO mice demonstrate attenuated neutrophilic, but regular eosinophilic response in HDM-induced asthma model. **(A)** Total number of cells in BAL fluid and the frequency (%) of eosinophils (CD45^+^SiglecF^+^CD11c^−^) and neutrophils (CD45^+^Ly6G^+^CD11b^+^) in the BAL of WT, IL-6 KO, and CD11c-IL-6 KO mice assessed by flow cytometry. **(B)** Frequency (%) of Th2 cells (CD45^+^CD3^+^CD4^+^CD8^−^ST2^+^) in the spleens of WT, IL-6 KO and CD11c-IL-6 KO mice assessed by flow cytometry. **(C)** Serum was collected 24 h after the last HDM challenge and the IgE levels in serum were determined by ELISA. **(D)** Quantitative RT-PCR analysis of *Ifng* and *Il17a* mRNAs in lungs 48 h after the last HDM challenge. Expression of each gene was normalized to *Actb*. Datasets were first tested for Gaussian distribution with the D'Agostino & Pearson omnibus normality test. Parametric or non-parametric One-way ANOVA with multiple comparisons tests were applied where appropriated. Data represent means ± SEM, 4–16 mice in each group. NS, not significant.

To specifically address the role of IL-6 produced by dendritic cells in allergic airway inflammation, we used mice with tissue-restricted inactivation of IL-6 in CD11c^+^ cells (CD11c-IL-6 KO) (23) and subjected them, together with IL-6 KO mice and littermate WT control mice, to HDM-induced asthma model (Figure 5). In CD11c-IL-6 KO mice, CD11c-driven *Cre* expression results in deletion of IL-6 in dendritic cells (23).

We found that ablation of IL-6 in dendritic cells reduced lymphocyte infiltration in the respiratory tract of experimental animals as compared to their littermate WT controls (Figure 5A). We then examined whether the deficit in IL-6 production by dendritic cells may affect the number of pathogenic cells, i.e., eosinophils, in the lungs. Surprisingly, flow cytometry analysis of bronchoalveolar lavage revealed that eosinophil accumulation in the BAL fluid did not differ from that found in WT mice (Figure 5A). Moreover, Th2 accumulation in the spleens was not reduced in CD11c-IL-6 KO mice, in contrast to Mlys-IL-6 KO mice (Figures 4B, 5B). IgE levels in BALF were reduced both in CD11c-IL-6 KO and Mlys-IL-6 KO, compared to WT mice (Figure 4C). These results indicate that IL-6 from dendritic cells does not contribute to the Th2 accumulation and eosinophilic inflammatory response during asthma.

To further investigate the role of IL-6 from dendritic cells in atopy during asthma, we analyzed systemic inflammatory response by determining IgE concentration in the serum and BAL fluid of the experimental animals (Figure 4C). IgE production in CD11c-IL-6 KO mice did not differ from that in WT mice, while in mice with complete knockout of IL-6 and Mlys-IL-6 KO mice IgE production was significantly lower. To establish whether IL-6 from dendritic cells affects neutrophilic inflammatory response (Figure 2, right panel), we compared neutrophil infiltration in the BAL fluid of CD11c-IL-6 KO mice, as well as, of mice with complete IL-6 deficiency and WT mice after HDM administration. We found that both CD11c-IL-6 KO mice and IL-6 KO mice had attenuated number of neutrophils in BAL fluid after HDM exposure (Figure 5A). In contrast, Mlys-IL-6 KO mice had similar numbers of neutrophils in the BAL fluid as compared to WT mice (Figure 4A). Furthermore, inhibition of neutrophilic inflammation in CD11c-IL-6 KO mice was associated with a marked decrease in mRNA (Figure 5D) and protein (Figure 5C) levels of IFNγ, which is a critical regulator of respiratory neutrophilia. The expression of IL-17A, which is crucial for development of neutrophilic asthma (39), was significantly diminished in the lungs of CD11c-IL-6 KO mice as compared to WT control mice (Figure 5D). Finally, mice with IL-6 deficiency in dendritic cells displayed diminished Th17, but not Th2, inflammatory response (Supplementary Figure 4) with abrogated neutrophilic accumulation in the lungs. These observations indicate that, although IL-6 produced by dendritic cells is necessary for the development of neutrophilic inflammatory response, it does not affect eosinophilia in response to HDM administration.

## Discussion

Asthma is a complex heterogeneous disease, with many subtypes that differ in etiology, severity, and treatment strategies (40). Strict division of this disease into endotypes is challenging, since clearly-defined endotypes of asthma are extremely rare, and more often intermediate forms with prevalence of some modality are observed. The association between the symptoms of allergic asthma and increased expression of IL-6 in patients was documented a long time ago (41). In mice, anti-IL-6 therapy of high-dose HDM asthma is effective (24), but the contribution of IL-6 to a more clinically relevant low-dose HDM (10 μg) asthma model was not experimentally determined. It should be noted that the inflammatory process in mouse models of HDM-induced asthma largely depends on the allergen dose and the mode of its administration. The preferred option with regard to clinical relevance is multiple administration of small doses of the allergen, rather than single injection of high dose HDM (42). High dose of HDM (more than 12.5 μg/kg) significantly increases the number of BALF lymphocytes and neutrophils compared to saline-challenged controls without the need for prior allergen sensitization. In contrast, a low dose i.n. HDM challenge (1.25 μg/kg) in allergen sensitized mice causes a significant increase in BALF of eosinophil, lymphocyte and neutrophil numbers. Thus, the low dose HDM protocol results in induction of sub-maximal levels of cellular inflammation in the BALF and is associated with an influx of eosinophils, lymphocytes and macrophages without an accompanying non-allergic cellular inflammation. In the present study, we selected an acute (2-week-long) HDM-induced asthma mouse model, driving allergen-induced inflammation without the undesirable impact from non-specific inflammatory response, which is relevant to eosinophilic endotype of severe asthma in humans.

Our results showed that ablation of IL-6 signaling as seen in IL-6 KO mice abrogated the increase in granulocyte and Th2 cell numbers in the airways (Figure 1B), secretion of IgE in BALF and serum (Figure 1C), expression of asthma-associated genes, such as *Tslp, Tgfb1, Muc5ac, Gob5, Il4* (Figures 1D,E), and mucus production in the lungs (Supplementary Figure 3) in the low-dose HDM-induced asthma. Moreover, the number of leukocytes both in BAL fluid and in lung tissue was significantly decreased in IL-6 KO mice. These findings indicate that in the context of acute asthma mouse model, IL-6 is one of the key regulatory cytokines modulating the immune response. IL-6 is known for its capability to promote the differentiation of Th2 cells, inhibit Th1 and Treg differentiation and expansion in response to allergen, activate immunoglobulin class-switching in plasma cells and enhance the differentiation of Th17 cells, which are engaged in severe asthma (11). Pharmacological blockade of IL-6 results in the reduction of airway inflammation in some asthma models (24), suggesting that this cytokine may be driving several types of responses to the allergen. However, the exact mechanism of IL-6 involvement in the pathogenesis of allergic asthma has not been determined previously, and the IL-6-producing cell types that make the most significant contribution to the development of airway hyper-reactivity and inflammation have not been established.

Although macrophages and neutrophils were considered as the predominant source of sIL-6R (43), both human and mouse CD4^+^ T-cells can also be the source of sIL-6R upon activation (44). CD4^+^ T-cells may, therefore, contribute to the development and progression of asthma by providing sIL-6R to cells initially non-responsive to IL-6. An important role of IL-6 in regulation of effector CD4^+^ T-cell fate (45) was attributed to driving IL-4 production during Th2 differentiation, inhibiting Th1 differentiation and, in synergy with TGFβ, promoting Th17 cell differentiation. Thus, CD4^+^ T cells can produce IL-6 as an autocrine regulator.

IL-6 is also produced by primary lung epithelial cells in response to a variety of different cell stress or damage signals (e.g., UV, irradiation, ROS, microbial products, viruses, or other proinflammatory cytokines). A number of studies have demonstrated overexpression of IL-6 by bronchial epithelial cells in patients with asthma, both in adults and children (46). A recent study in mouse model of asthma confirmed an important contribution of IL-6 produced by epithelial cells in the pathogenesis of aspergillus-induced asthma. It was shown that high levels of IL-6 mRNA were constitutively present in mouse primary lung epithelial cells, but not in lung resident immune cells. Moreover, direct interaction of fungal β-glucans with lung epithelial cells triggered IL-6 production by lung epitheliocytes (47). In addition to constitutive expression of IL-6 by lung epithelial cells in steady state, the exposure to allergen can further induce production of this cytokine prior to the recruitment of inflammatory cells. Thus, the presence of IL-6 in the airways of asthmatic patients may not be the result of ongoing inflammation, but rather due to the “activated state” of pulmonary epithelial cells. Finally, IL-6 production is characteristic for lung fibroblasts upon activation (48).

In the present study we demonstrate that, both in steady state conditions and following acute asthma induction with low-dose HDM, the vast majority of IL-6 positive cells are leukocytes. Moreover, the number of IL-6^+^CD45^+^ cells was significantly increased in asthmatic as compared to healthy mice. At steady state, most of the IL-6-producing cells were represented by macrophages and monocytes, whereas after the disease induction, the contribution from IL-6-producing T-cells increased (Figure 3D). We, thus, hypothesized that myeloid cells are the source of disease-triggering pathogenic IL-6, which stimulates the development of the inflammatory responses in the respiratory tract, while the increase in the proportion of lymphoid cells in the pool of IL-6-producing cells may be a secondary event. Additionally, taking into account that alveolar macrophages and dendritic cells may produce IL-6 with different kinetics (49), we hypothesized that the effects of IL-6 deficiency in macrophages and dendritic cells on allergic airway inflammation may differ.

Increasing evidence suggests that macrophage-derived IL-6 plays the essential role in Th2-mediated allergic response. It was previously shown that IL-6 can enhance the polarization of alternatively activated macrophages (50) in synergy with IL-4 and IL-13, the two differentiation factors for alternatively activated macrophages which also play a pivotal role in eosinophilic allergic inflammation. Interestingly, the observed effect of IL-6 on macrophage polarization was partially dependent on up-regulation of the IL4Ra chain, which is consistent with the previously published data, showing that transfer of IL-4Ra^+^ macrophages is sufficient to enhance Th2-driven eosinophilic allergic inflammation in the lungs (51). Moreover, HDM treatment induces alternatively activated macrophage polarization and Th2-mediated eosinophilia in the lungs (52). Finally, it was recently reported that inhibition of alternatively activated macrophage polarization in HDM-induced asthma model leads to reduced eosinophilic inflammatory response and subsequent shift toward neutrophilic inflammation (53). Since macrophages constitute the predominant fraction of IL-6 producing cells in the lungs (Figure 3D), we evaluated the contribution of IL-6 from macrophages to the development of allergic asthma using reverse genetics approach. In the low-dose (10 μg) HDM-induced acute asthma model, we found that cell type-restricted deletion of IL-6 in macrophages leads to significant reduction of eosinophilic inflammation in the lungs (Figure 4A) and to attenuation of Th2 accumulation in the periphery (Figure 4B). Moreover, IgE production in Mlys-IL-6 KO mice was markedly decreased as compared to WT control mice (Figure 4C). Our results indicate that IL-6 from macrophages promotes Th2-driven eosinophilic inflammation during HDM-induced asthma, probably, due to IL-6-mediated macrophage polarization toward the alternatively activated macrophages. Additionally, we found that Th2, but not Th17, response was ablated in mice with IL-6 deficiency restricted to macrophages (Supplementary Figure 4). These data provide further support for important role of macrophages in the development of eosinophilic inflammatory response to the allergen and suggests that this impact involves IL-6.

Previous studies using an adoptive transfer of IL-6 deficient dendritic cells to WT mice, indicated the role of dendritic-cell-derived IL-6 in allergic inflammation, characterized by increased Th2 response and increased eosinophilia (25). In contrast, in another study, IL-6 produced by dendritic cells was shown to inhibit Th2 inflammatory response (54). We, thus, expected that IL-6 from dendritic cells may be the main factor determining the severity of Th2 responses to HDM in allergic asthma model. However, eosinophilic, Th2-mediated responses in mice with IL-6 deficiency in dendritic cells showed only a modest decrease. Neither lung eosinophilia (Figure 5A), nor the accumulation of Th2 cells in the periphery (Figure 5B) was significantly different from that in the WT control group. Unexpectedly, we found that neutrophilic response to HDM in CD11c-IL-6 KO mice was significantly reduced (Figure 5A), as well as, the production of IFNγ (Figure 5C) and expression level of IL-17A in the lungs (Figure 5D). Of note, recent findings highlighted the crucial role of dendritic cell-derived IL-6 in the development of experimental encephalomyelitis through the process called trans-presentation (55) that may be a distinctive feature of this cellular source of IL-6. T cells are able to recognize IL-6 produced by dendritic cells and respond by specific signaling cascade leading to STAT activation and differentiation into Th17 subsets. This data supports our observation that mice with IL-6-deficiency in dendritic cells display decreased Th17, but not Th2 inflammation (Supplementary Figure 4) with abrogated neutrophilic response in the lungs. Neutrophilic asthma is characterized by increased Th1 and Th17 cell responses with release of cytokines from Th1 and Th17 cells and by neutrophil recruitment to the site of inflammation (32, 33). Moreover, severity of asthma correlates with the level of IL-17A in the lungs, sputum and BALF (33). In patients with neutrophilic asthma driven by Th1/Th17 responses the release of IL-6, IL-17, IL-8, and IFNγ is associated with more severe refractory steroid-resistant endotype. However, randomized studies of Brodalumab, a human monoclonal antibody against IL-17a, did not show significant effects in treatment of severe asthma (56). Clinical trials of Suricumab, a fully human monoclonal antibody against IL-6, are currently in progress and therapeutic efficacy of systemic IL-6 blockade for severe asthma is yet to be determined. Nevertheless, the concept of severe asthma treatment is changing from one-drug-fits-all approach to more specific endotype-dependent therapy. In addition, the idea of cell-type-specific anti-cytokine therapy is currently being evaluated in several autoimmune disease models (57, 58). Our findings suggest that IL-6 from dendritic cells may contribute to the development of neutrophilic asthma, and that IL-6 expression by dendritic cells may lead to exacerbation of allergic inflammation in the lungs. Considering the high heterogeneity of dendritic cells and macrophages in the lungs, further studies will be required to dissect the role of individual IL-6 producing myeloid cell populations in lung inflammation and airway hyper-responsiveness.

**Figure 6:**
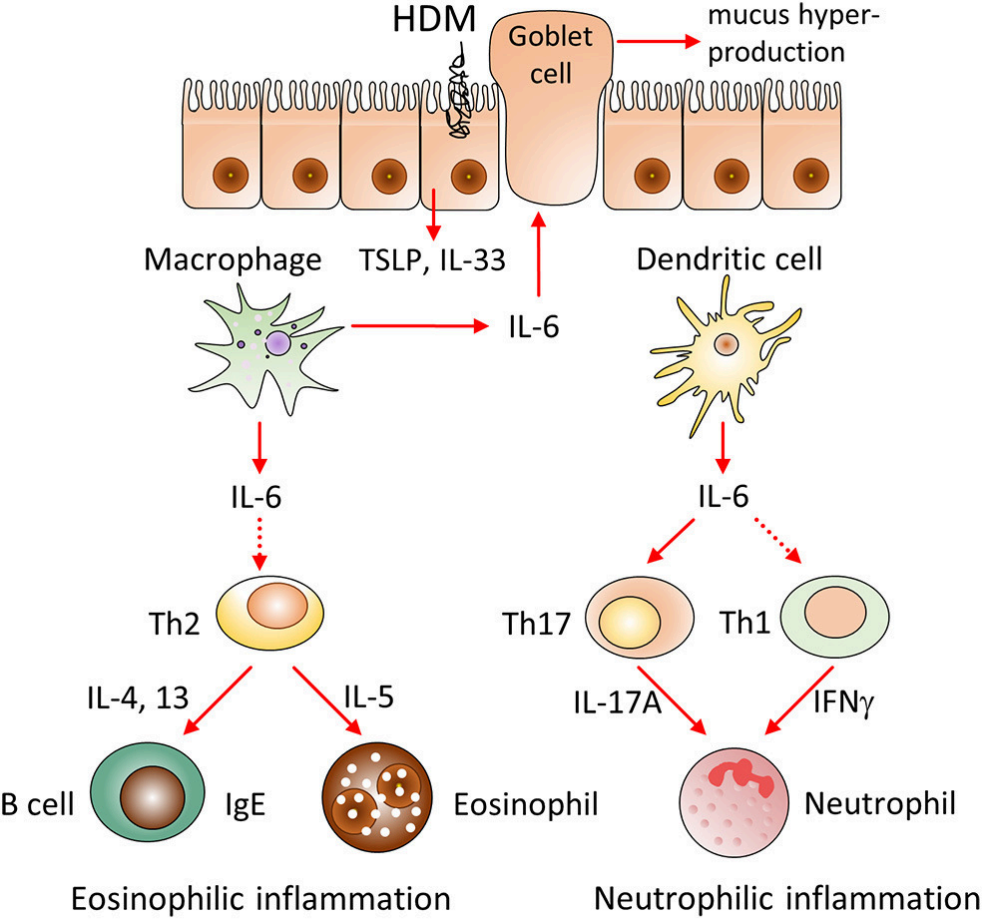
Hypothetical model of the involvement of IL-6 from different cellular sources in the pathogenesis of HDM-induced allergic asthma. We propose that IL-6 produced by macrophages and dendritic cells distinctively promotes HDM-induced airway inflammation. IL-6 produced by macrophages contributes to type 2 allergic inflammation, including eosinophil accumulation, IgE production and mucus hypersecretion via induction of Th2 cells differentiation and production of IL-33, TSLP, IL-4, IL-13 cytokines. In contrast, IL-6 from dendritic cells induces pathogenic accumulation of neutrophils via induction of IL-17A and IFNγ, produced by Th17 and Th1 subsets. Therefore, selective targeting of IL-6 in macrophages or dendritic cells can be beneficial in corresponding eosinophilic and neutrophilic asthma endotypes.

Taken together, our results demonstrate the pathogenic role of IL-6 in clinically relevant low-dose acute HDM-induced asthma model and reveal the distinct roles of IL-6 produced by macrophages and dendritic cells in acute allergic airway inflammation. Whereas, IL-6 from macrophages contributes to type 2 allergic inflammation, IL-6 from dendritic cells is critical for induction of neutrophilic inflammation (Figure 6). Therefore, the approach of cell type-restricted targeting of IL-6 may be effective in the treatment of allergic asthma, especially its severe neutrophilic type.

## Author contributions

EOG, EAG, ON, and RZ performed experiments. JH provided critical materials. EOG, MD, AT, and SN designed experiments, discussed results and wrote the manuscript.

### Conflict of interest statement

The authors declare that the research was conducted in the absence of any commercial or financial relationships that could be construed as a potential conflict of interest.

## Supplementary material

Supplementary Figure 1 Asthma-associated gene expression and cytokine production in IL-6 KO and WT mice 48 h after last HDM challenge. **(A)** Quantitative RT-PCR analysis of *Muc5b, Ifng, Il5, Il1b, Il33*, and *Il10* mRNAs in lungs. Expression of each gene was normalized to *Actb*. **(B)** Serum was collected 24 h after the last HDM challenge. IFNγ levels in BALF and IL-1β levels in serum were determined by ELISA. Datasets were first tested for Gaussian distribution with the D'Agostino & Pearson omnibus normality test. Parametric or non-parametric comparison tests were applied where appropriated. Data represent means ± SEM, 5–17 mice in each group.

Supplementary Figure 2 Gating strategy for different IL-6 producing cell populations. **(A)** Lungs from WT mice 48 h after last HDM challenge were subjected to enzymatic digestion to generate single cell suspensions of tissue samples. After that cells were stimulated with PMA and ionomycin in a humidified atmosphere containing 5% CO_2_ at 37°C for 4 h. Brefeldin A was used to block protein Golgi transport. Distinct populations of leukocytes were distinguished with the following Ab panel: Fixable Viability Dye-eFluor 780, anti-CD45 (30-F11), anti-Ly6G (RB6-8C5), anti-CD103 (2E7), anti-CD11b (M1/70), anti-CD11c (N418), anti-SiglecF (1RNM44N), anti-CD4(GK1.5, eBioscience), anti-CD8(53-6.7, BioLegend), anti-ST2(RMST2-2, eBioscience), anti-CD19 (6D5, eBioscience) conjugated with FITC, PE, APC, PE-Cy7, PerCP-Cy5.5, and AmCyan. Cells were fixed and stained with anti-IL-6 PB-conjugated antibody. Mouse IgG1 Ab -PB (MOPC-21, eBioscience) was used as isotype control. **(B)** IL-6 positive cells were gated on the basis of isotype and FMO controls **(C)** Representative percentage ratio of IL-6 producing cells of different lung leukocyte populations in steady state (left) and 48 h after last HDM introduction (right) in WT mice assessed by flow cytometry. Data represent means ± SEM, 5–11mice in each group.

Supplementary Figure 3 Histological assessment of lung inflammation on PAS-stained lung sections from WT, CD11c-IL-6 KO, MLys-Cre-IL-6 KO, and IL-6 KO mice. Red arrow denotes goblet cells in WT, CD11c-IL-6 KO, MLys-Cre-IL-6 KO lung tissue stained with Periodic acid-Schiff (PAS) staining.

Supplementary Figure 4 Mlys-IL-6 KO mice demonstrate a decrease in frequency of Th2 cells, whereas CD11C-IL-6 KO mice show a reduction of Th17 cells in lungs in HDM-induced asthma. **(A)** Frequency (%) of activated Th2 cells (CD45^+^ CD3^+^ CD4^+^ CD8^−^ ST2^+^ IL-4^+^), Th1 cells (CD45^+^ CD3^+^ CD4^+^ CD8^−^ INFg^+^ TNF^+^), T reg (CD45^+^ CD3^+^ CD25^+^ FoxP3^+^ HELIOS^+^) and Th17 cells (CD45^+^ CD3^+^ CD4^+^ CD8^−^ IL-17A^+^ RORyt^+^) in the lungs of WT and Mlys-IL6 KO mice assessed by flow cytometry. **(B)** Frequency (%) of activated Th2 cells (CD45^+^ CD3^+^ CD4^+^ ST2^+^ IL-4^+^), Th1 cells (CD45^+^ CD3^+^ CD4^+^ INFg^+^ TNF^+^), T reg (CD45^+^ CD3^+^ CD4^+^ FoxP3^+^ HELIOS^+^) and Th17 cells (CD45^+^ CD3^+^ CD4^+^ IL-17A^+^ RORyt^+^) in the lungs of WT and CD11c-IL6 KO mice assessed by flow cytometry. Datasets were first tested for Gaussian distribution with the D'Agostino & Pearson omnibus normality test and parametric comparison test (*t*-test) was applied. Data represent means ± SEM, 5–8 mice in each group. ^*^*P* < 0.05.
