# Supplementary figures and images for: Non-redundant Functions of IL-6 Produced by Macrophages and Dendritic Cells in Allergic Airway Inflammation
Source: Front Immunol. 2018 Nov 26;9:2718. doi: 10.3389/fimmu.2018.02718 (PMC6276801; doi:10.3389/fimmu.2018.02718)

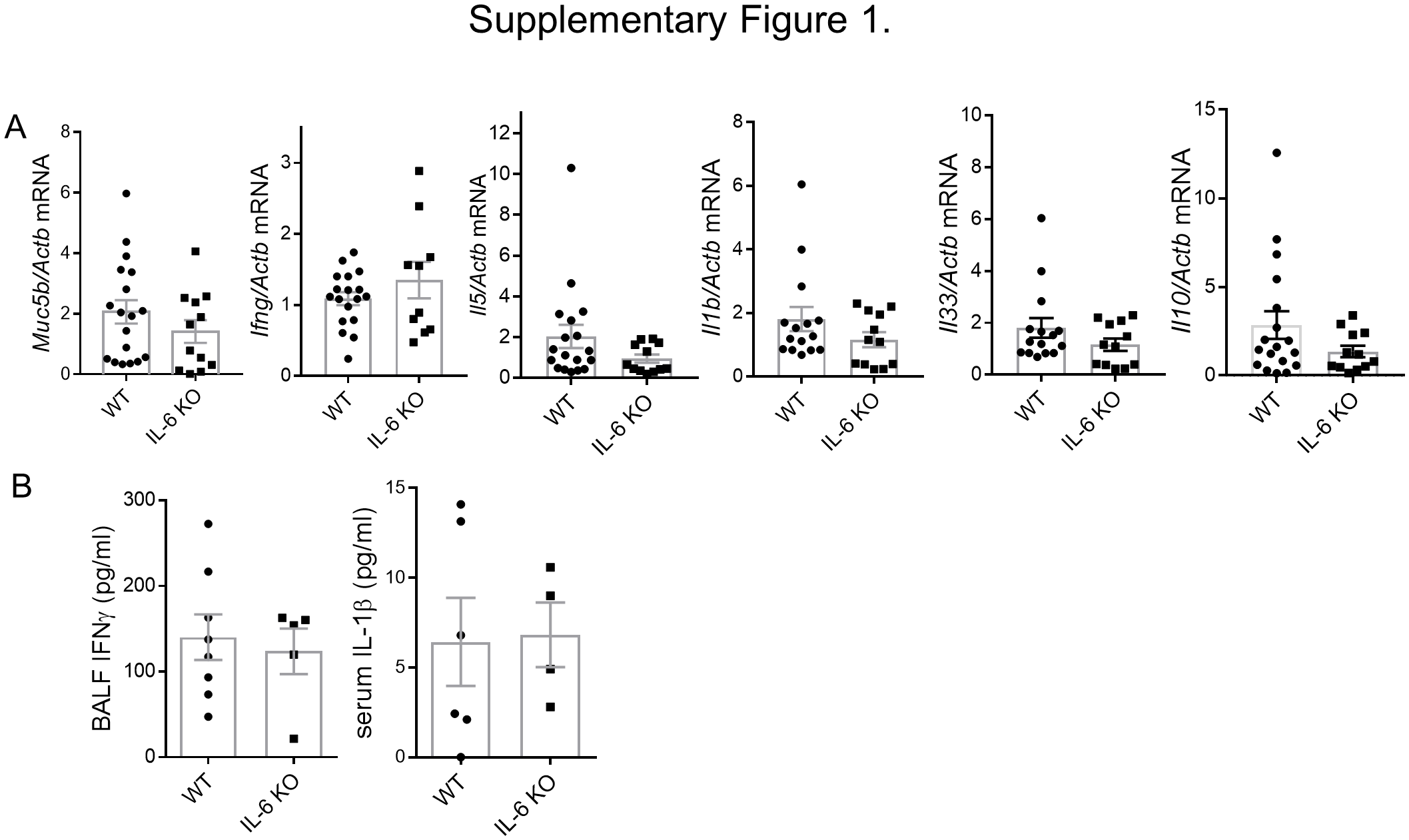

Supplement: Supplementary Figure 1 — Asthma-associated gene expression and cytokine production in IL-6 KO and WT mice 48 h after last HDM challenge. (A) Quantitative RT-PCR analysis of Muc5b, Ifng, Il5, Il1b, Il33, and Il10 mRNAs in lungs. Expression of each gene was normalized to Actb. (B) Serum was collected 24 h after the last HDM challenge. IFNγ levels in BALF and IL-1β levels in serum were determined by ELISA. Datasets were first tested for Gaussian distribution with the D'Agostino & Pearson omnibus normality test. Parametric or non-parametric comparison tests were applied where appropriated. Data represent means ± SEM, 5–17 mice in each group. [file Image_1.tif]

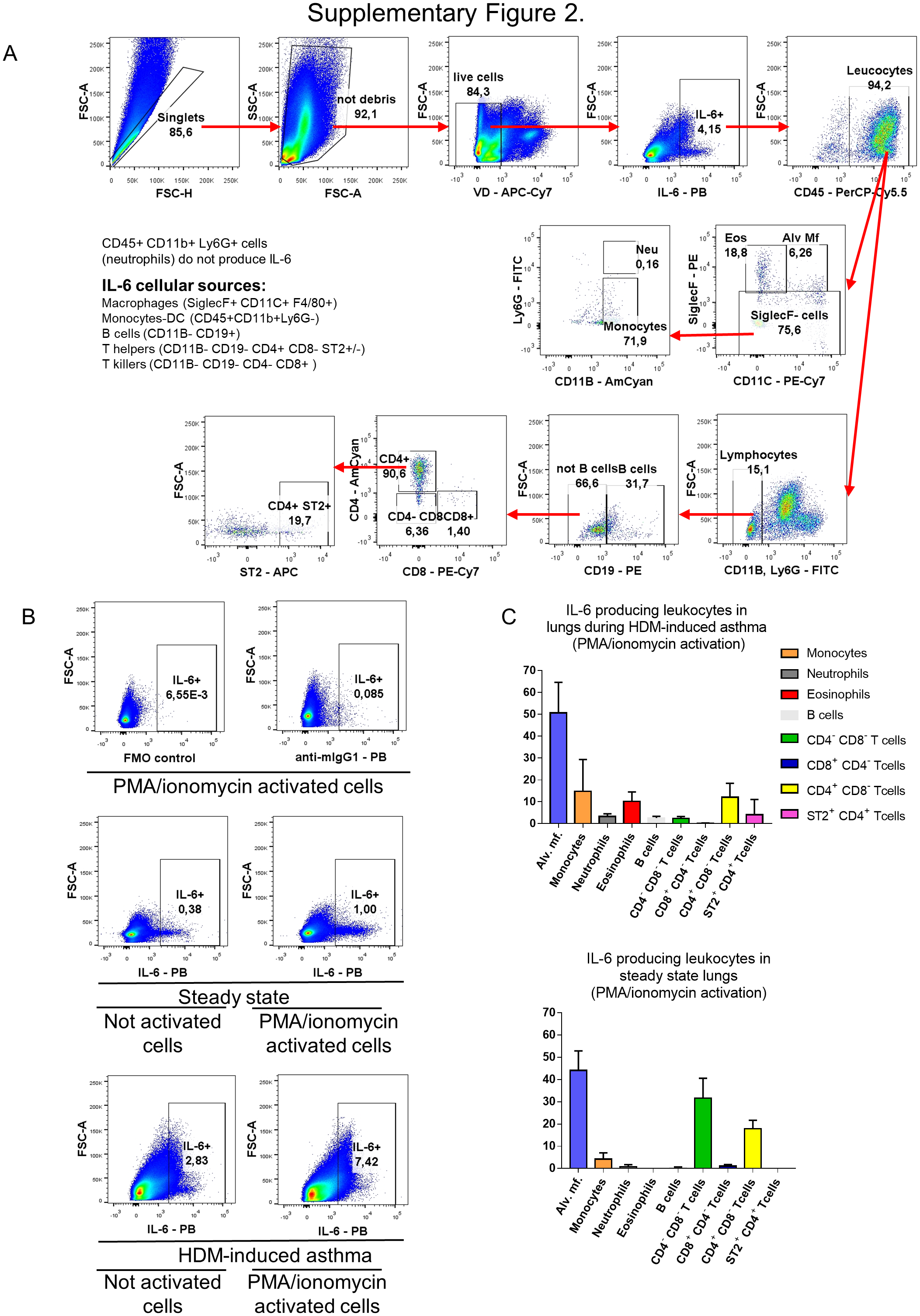

Supplement: Supplementary Figure 2 — Gating strategy for different IL-6 producing cell populations. (A) Lungs from WT mice 48 h after last HDM challenge were subjected to enzymatic digestion to generate single cell suspensions of tissue samples. After that cells were stimulated with PMA and ionomycin in a humidified atmosphere containing 5% CO2 at 37°C for 4 h. Brefeldin A was used to block protein Golgi transport. Distinct populations of leukocytes were distinguished with the following Ab panel: Fixable Viability Dye-eFluor 780, anti-CD45 (30-F11), anti-Ly6G (RB6-8C5), anti-CD103 (2E7), anti-CD11b (M1/70), anti-CD11c (N418), anti-SiglecF (1RNM44N), anti-CD4(GK1.5, eBioscience), anti-CD8(53-6.7, BioLegend), anti-ST2(RMST2-2, eBioscience), anti-CD19 (6D5, eBioscience) conjugated with FITC, PE, APC, PE-Cy7, PerCP-Cy5.5, and AmCyan. Cells were fixed and stained with anti-IL-6 PB-conjugated antibody. Mouse IgG1 Ab -PB (MOPC-21, eBioscience) was used as isotype control. (B) IL-6 positive cells were gated on the basis of isotype and FMO controls (C) Representative percentage ratio of IL-6 producing cells of different lung leukocyte populations in steady state (left) and 48 h after last HDM introduction (right) in WT mice assessed by flow cytometry. Data represent means ± SEM, 5–11mice in each group. [file Image_2.tif]

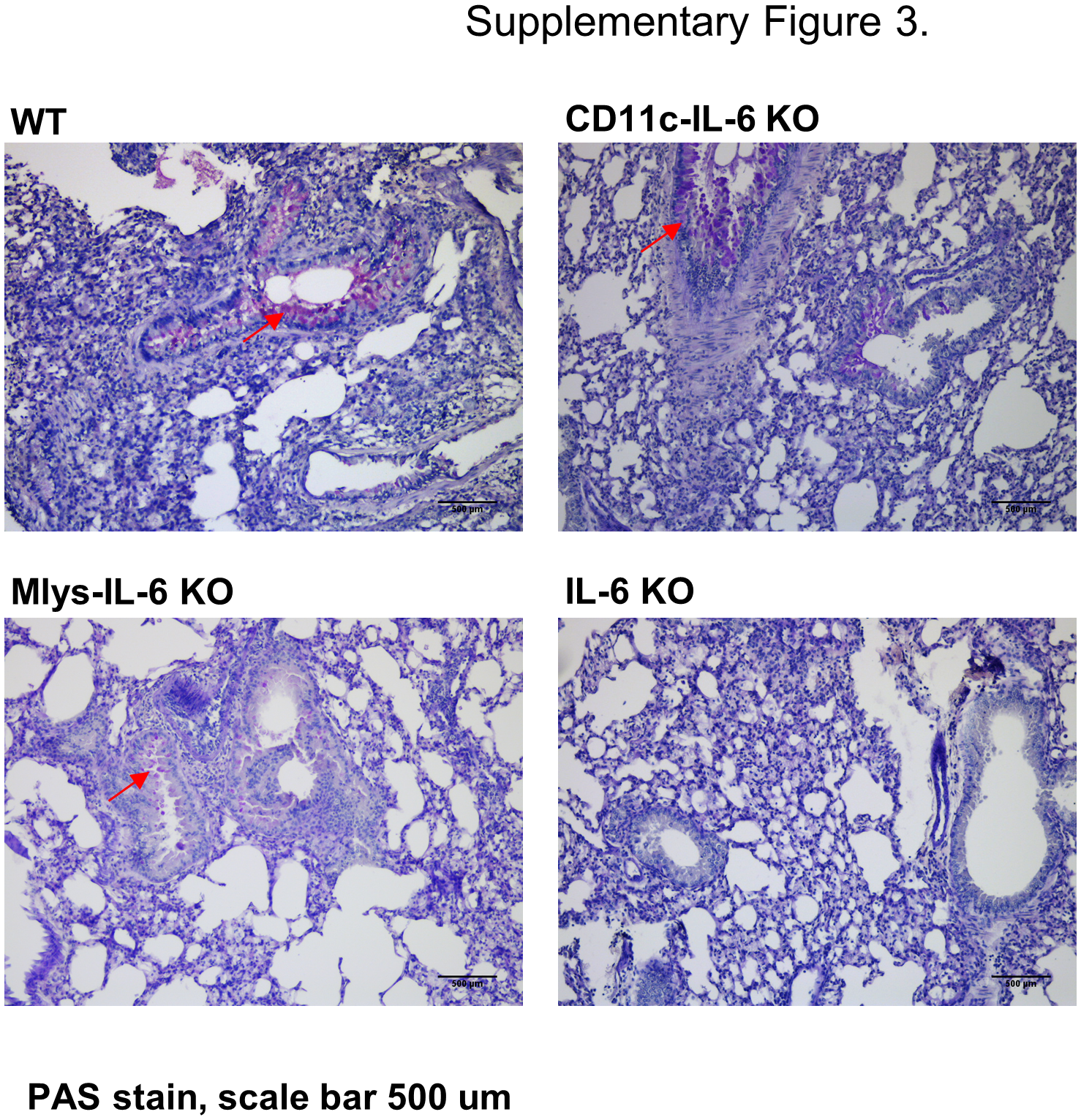

Supplement: Supplementary Figure 3 — Histological assessment of lung inflammation on PAS-stained lung sections from WT, CD11c-IL-6 KO, MLys-Cre-IL-6 KO, and IL-6 KO mice. Red arrow denotes goblet cells in WT, CD11c-IL-6 KO, MLys-Cre-IL-6 KO lung tissue stained with Periodic acid-Schiff (PAS) staining. [file Image_3.tif]

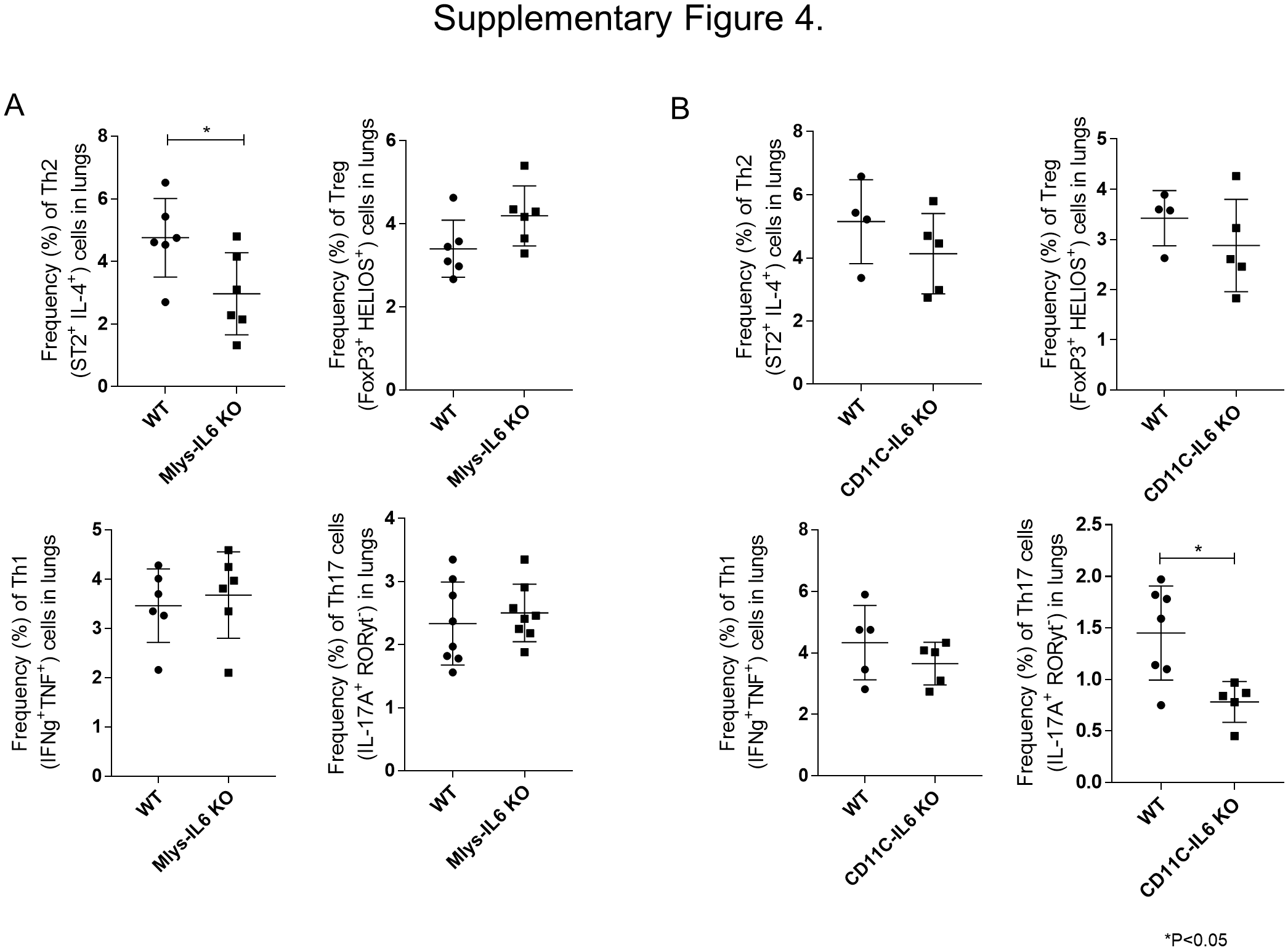

Supplement: Supplementary Figure 4 — Mlys-IL-6 KO mice demonstrate a decrease in frequency of Th2 cells, whereas CD11C-IL-6 KO mice show a reduction of Th17 cells in lungs in HDM-induced asthma. (A) Frequency (%) of activated Th2 cells (CD45+ CD3+ CD4+ CD8− ST2+ IL-4+), Th1 cells (CD45+ CD3+ CD4+ CD8− INFg+ TNF+), T reg (CD45+ CD3+ CD25+ FoxP3+ HELIOS+) and Th17 cells (CD45+ CD3+ CD4+ CD8− IL-17A+ RORyt+) in the lungs of WT and Mlys-IL6 KO mice assessed by flow cytometry. (B) Frequency (%) of activated Th2 cells (CD45+ CD3+ CD4+ ST2+ IL-4+), Th1 cells (CD45+ CD3+ CD4+ INFg+ TNF+), T reg (CD45+ CD3+ CD4+ FoxP3+ HELIOS+) and Th17 cells (CD45+ CD3+ CD4+ IL-17A+ RORyt+) in the lungs of WT and CD11c-IL6 KO mice assessed by flow cytometry. Datasets were first tested for Gaussian distribution with the D'Agostino & Pearson omnibus normality test and parametric comparison test (t-test) was applied. Data represent means ± SEM, 5–8 mice in each group. *P < 0.05. [file Image_4.tif]
